# Individualized strategies for depression: narrative review of clinical profiles responsive to vortioxetine

**DOI:** 10.1186/s12991-024-00505-1

**Published:** 2024-05-16

**Authors:** Alessandro Cuomo, Andrea Aguglia, Domenico De Berardis, Antonio Ventriglio, Camilla Gesi, Andrea Fagiolini

**Affiliations:** 1 Department of Molecular and Developmental Medicine, University of Siena School of Medicine, Siena, Italy; 2 Department of Neuroscience, Rehabilitation, Ophthalmology, Genetics, Maternal and Child Health, Section of Psychiatry, University of Genoa, Genoa, Italy; 3 IRCCS Ospedale Policlinico San Martino, Genoa, Italy; 4 Department of Mental Health, ASL 4, Teramo, 64100 Italy; 5 Department of Clinical and Experimental Medicine, University of Foggia, Foggia, Italy; 6 Department of Mental Health and Addiction, ASST Fatebenfratelli-Sacco, Milan, Italy

**Keywords:** Vortioxetine, Depression, Cognitive dysfunction, Anhedonia, Emotional blunting, Multimodal Antidepressant, Individualized treatment, Comorbid anxiety symptoms

## Abstract

**Background:**

Depression is a highly heterogeneous disorder, often resulting in suboptimal response and remission rates. This underscores the need for more nuanced clinical characterization of patients to tailor individualized treatment plans. Emerging evidence highlights the critical role of cognitive and emotional dysfunction in major depression, prompting the exploration of novel therapeutic interventions that target these specific symptom domains.

**Main text:**

Vortioxetine, a multimodal antidepressant, enhances serotonergic activity while also modulating several other neurotransmitter systems involved in depressive symptoms such as emotional blunting, anhedonia, and cognitive dysfunction. Numerous randomized, placebo-controlled trials have demonstrated vortioxetine’s efficacy and safety in treating depression, particularly in specific subgroups of depressed patients, including those with cognitive deficits and comorbid anxiety symptoms or disorders. Although not randomized or placebo-controlled, studies have also shown vortioxetine’s efficacy in depressed patients with emotional blunting or anhedonia. Vortioxetine’s ability to effectively treat a range of depressive symptoms, including anhedonia, emotional blunting, anxiety, and cognitive dysfunction, provides an individualized treatment solution for depressed individuals suffering from these symptoms. The purpose of this paper is to identify clinical profiles of patients who may benefit from vortioxetine, with the goal of optimizing therapeutic outcomes.

**Conclusion:**

Vortioxetine has been shown to be effective for patients with depression and symptoms such as anhedonia, emotional blunting, anxiety, and cognitive dysfunction. Tailoring treatment plans to individual needs and personalizing treatment choices based on the specific symptoms presented by depressed patients improve treatment outcomes.

## Background

The treatment of depression presents several challenges: (a) Varying contributions of biological, psychological, and environmental factors in each individual making it difficult to identify the specific cause and necessitating treatments that address multiple factors [1, 2]. (b) Stigma and barriers to help-seeking behavior [3]. (c) Individual variability, as depression manifests differently across individuals. There is no one-size-fits-all treatment, and what works for one person may not be as effective for another [4]. (d) Comorbidity with other mental or physical health conditions, complicating treatment and requiring a holistic approach [5]. (e) Treatment resistance [6]. (f) Limited access to mental health services particularly in low-income areas, leading to disparities in mental health care [7]. (g) Medication side effects, making it challenging to find the right balance between efficacy and tolerability [8, 9]. (h) Relapse prevention as maintenance and follow-up care are critical for sustaining long-term improvements in mental health [10]. (i) Lack of objective measures, unlike some physical illnesses, as there are no clear biomarkers or diagnostic tests for depression [11]. Diagnosis often relies on self-report and clinical assessment, making it difficult to objectively measure treatment progress. l) Patient engagement and adherence, including motivating individuals to actively participate in their treatment consistently take medications and make lifestyle changes.

Addressing depression’s multifaceted challenges requires a comprehensive and multidisciplinary approach involving mental health professionals, support networks, and policy changes to improve access to mental health care [12]. Ongoing research and advancements in understanding depression are crucial for developing more effective and personalized treatment strategies. Major depressive disorder (MDD) treatment challenges include delayed pharmacotherapy response, adverse effects, and persistent cognitive dysfunction affecting executive function, memory, and attention, which can hinder recovery even during remission [13, 14].

MDD’s complexity may benefit from personalized clinical approaches [15, 16], yet current treatment choices are often based on trial-and-error strategies, leading to a lack of remission in many cases and treatment resistance in about 30% of patients [16].

Vortioxetine, approved for MDD, offers a unique mechanism of action, modulating neurotransmission in multiple systems [17].

This narrative review explores the impact of vortioxetine on various clinical presentations of patients with MDD who may benefit from this medication.

## Materials and methods

For the purposes of this expert opinion paper, we appraised and selected studies to report and comment on the existing literature that we considered most relevant for a qualitative summary and interpretation of our perceived perspectives on the impact of vortioxetine on the treatment armamentarium for major depressive disorder. As a narrative review, this manuscript is primarily based on articles selected according to our personal knowledge, experience, and perspective, with the goal of sharing our clinical or research experiences and perspectives.

We conducted a search strategy in PubMed to identify relevant studies using the following search string: ((vortioxetine[title/abstract]) AND (major depressive disorder[title/abstract] OR depression[title/abstract])) AND (efficacy[title/abstract] OR tolerability[title/abstract] OR adverse effects[title/abstract] OR symptoms[title/abstract]). We read the titles and abstracts of the 314 papers resulting from our search strategy. The full text of 41 papers considered most relevant to the topic of this paper were retrieved from PubMed or other databases, analyzed, and discussed in this paper. We maintained a narrative review approach, as 10 additional papers were retrieved from the references of the above papers and included in our critical review.

### Pharmacological profile of vortioxetine

Vortioxetine, approved for MDD in adults, is administered at doses ranging from 5 to 20 mg/day, with a recommended starting dose of 10 mg/day for those under 65 and 5 mg/day for older adults [13]. This antidepressant is distinguished by its multimodal mechanism of action, influencing various neuroreceptor systems [18]. Originating from a drug discovery program, vortioxetine’s development was inspired by hypotheses combining SERT inhibition and 5-HT_1A_ receptor modulation [19]. Classified under a new system for psychotropic medications, vortioxetine uniquely combines serotonin (5-HT) receptor activity modulation with SERT inhibition across its dosage spectrum [13, 19]. Its action is primarily through serotonergic receptor modulation and SERT inhibition [20]. Preclinical studies reveal vortioxetine’s broad receptor activity, including antagonism at 5-HT_3_, 5-HT_7_, and 5-HT_1D_ receptors, partial agonism at 5-HT_1B_ receptors, and agonism at 5-HT_1A_ receptors, alongside SERT inhibition. This extensive modulation impacts neurotransmission across multiple systems, including serotonin, norepinephrine, dopamine, histamine, acetylcholine, GABA, and glutamate, contributing to its antidepressant and anxiolytic effects, as well as cognitive function enhancement observed in animal studies [21]. Vortioxetine’s SERT saturation is dose-dependent, contrasting with selective serotonin reuptake inhibitors (SSRIs) and serotonin norepinephrine reuptake inhibitors (SNRIs). Positron emission tomography (PET) studies using 5-HT transporter ligands (11 C-MADAM or 11 C-DASB) demonstrated that 5-HT transporter occupancy in the raphe nuclei is around 50% at 5 mg/day, 65% at 10 mg/day, and over 80% at 20 mg/day [22]. This is significant compared to SNRIs and SSRIs, where serotonin reuptake transporter occupancy is typically over 80% even at the lowest effective doses, explaining the limited efficacy increase but worsened tolerability profile at higher doses of these medications [23].

### Clinical profiles responsive to Vortioxetine

Clinical trials and meta-analyses have consistently demonstrated vortioxetine’s effectiveness in MDD. A meta-analysis of 11 randomized, placebo-controlled trials found significant reductions in Montgomery–Åsberg Depression Rating Scale (MADRS) scores across vortioxetine doses (5–20 mg/day), indicating a dose-dependent antidepressant effect [24]. Further, pooled analyses and studies have corroborated these findings, demonstrating greater efficacy and rapid symptomatic response at higher doses [25, 26].

Vortioxetine has been demonstrated to significantly improve most depressive symptoms, including cognitive impairment [27–39], anhedonia [40–42], and emotional blunting [43]. It has also proven efficacious in improving depressive symptoms in patients with comorbid anxiety [44–47], and psychiatric comorbidities such as early-stage dementia [46].

### Efficacy for patients with cognitive dysfunction

Vortioxetine’s pro-cognitive effects have been demonstrated in several studies. Animal and preclinical studies indicate its potential to enhance synaptic plasticity and cortical activity, impacting cognitive functions [27–29]. Clinical trials using cognitive assessments like the Rey Auditory Verbal Learning Test (RAVLT) and Digit Symbol Substitution Test (DSST) have shown significant improvements in cognitive performance in MDD patients treated with vortioxetine, independent of depressive symptom improvement [30, 31, 33]. These effects are particularly pronounced in working patients, highlighting vortioxetine’s role in improving work productivity and quality of life [34, 35, 48]. Meta-analyses further reinforce vortioxetine’s superiority in enhancing cognitive functions compared to other antidepressants [36–39]. In a study involving 100 patients with Alzheimer’s disease (AD) and depression, vortioxetine was not significantly superior to placebo in reducing depressive symptoms or cognitive impairment. However, overall tolerability and patient safety were similar to placebo. The authors concluded that additional studies are needed to replicate the efficacy and tolerability of vortioxetine in AD patients with depression [49]. The effectiveness and tolerability of vortioxetine in improving depressive symptoms, cognitive performance, functioning, and quality of life in patients with MDD and comorbid early-stage dementia, were studied in 82 patients who received vortioxetine for 12 weeks. A significant improvement was shown in depressive symptom severity, cognitive performance, functioning, and quality of life. Vortioxetine was well tolerated [46]. A prospective, randomized, 12 month, parallel-group study investigated the efficacy of vortioxetine compared to other conventional antidepressants on cognitive functions in AD patients with depressive symptoms. The study concluded that vortioxetine had a beneficial effect on cognition and mood in elderly AD patients and was safe and well tolerated [50].

### Efficacy for patients with anhedonia and emotional blunting

Vortioxetine has been shown to be effective in treating anhedonia, a core symptom of depression, as evidenced by improvements in the Snaith-Hamilton Pleasure Scale (SHAPS) and MADRS anhedonia factor scores [40, 41]. Its long-term efficacy in reducing anhedonia-related symptoms has been supported over 52 weeks of treatment [42]. In addition, vortioxetine has shown promise in alleviating emotional blunting in patients with MDD, particularly those with a partial response to SSRIs/SNRIs [43]. Although these studies were not randomized or placebo-controlled, improvements in anhedonia and emotional blunting were statistically significant.

### Efficacy for patients with comorbid anxiety

Vortioxetine effectively reduces anxiety symptoms in MDD, as demonstrated in a meta-analysis of trials with high baseline anxiety levels [44] and an update pooled analysis of fixed-dose studies [45].

The RECONNECT study and a subgroup analysis of the RELIEVE study further validated vortioxetine’s efficacy in patients with severe depression and comorbid generalized anxiety disorder (GAD) [46, 47]. Moreover, vortioxetine’s effectiveness extends to patients with MDD and comorbidities like alcohol use disorder and substance use disorder. In these populations, vortioxetine has been shown to significantly improve depressive symptoms and functional impairment [51, 52]. These findings highlight the potential of vortioxetine as a treatment option for patients with MDD and various comorbid conditions, particularly those involving anxiety and substance use disorders.

### Efficacy for patients with physical comorbidities

Vortioxetine has been shown to be well-tolerated and efficacious in patients with MDD and comorbid physical conditions, such as cardiovascular disease, diabetes mellitus, Parkinson’s disease [53] and, to a lesser extent, chronic obstructive pulmonary disorders (COPD) [54]. Notably, vortioxetine’s effectiveness and tolerability extend to older populations, who often deal with multiple comorbidities and polypharmacy [55]. This is particularly relevant, as the presence of physical comorbidities can complicate the management of MDD and negatively impact treatment outcomes.

Recent evidence suggests that vortioxetine may have beneficial effects beyond its direct impact on depressive symptoms. A recent meta-analysis [66] demonstrated that vortioxetine significantly improved physical symptoms associated with depression, such as pain, sleep disturbances, and somatic anxiety. The multimodal mechanism of action of vortioxetine, involving modulation of several neurotransmitter systems, may contribute to its ability to alleviate these somatic correlates of depression. Furthermore, preclinical studies have revealed that vortioxetine possesses anti-inflammatory and antioxidative properties. There is some evidence that vortioxetine exerts anti-inflammatory and immunomodulatory effects on human monocytes and macrophages, possibly through its actions on the serotonergic system and direct inhibition of cyclooxygenases [67]. These findings suggest that vortioxetine’s anti-inflammatory activity may be particularly relevant in the context of post-COVID-19 depression. Patients with post-COVID-19 major depressive episodes treated with vortioxetine experienced significant improvements in physical and cognitive symptoms, as well as a reduction in inflammatory markers [68]. Given the high prevalence and clinical implications of post-COVID-19 depression, further research on the potential benefits of vortioxetine in this population is warranted (Table 1).

**Table 1:** Summary of Vortioxetine clinical findings

Study	Vortioxetine Dosage	Patient Characteristics	Main Clinical Effects
Thase et al. (2016)	5–20 mg/day	Adults with MDD	Dose-dependent reduction in MADRS scores
Christensen et al. (2023a)	20 mg/day	Adults with MDD	Greater efficacy and rapid response at 20 mg/day
Christensen et al. (2023b)	20 mg/day	Adults with MDD	Clinical benefits at 20 mg/day
Dale et al. (2014)	-	Animals	Enhanced synaptic plasticity and cortical activity
Leiser et al. (2014)	-	Animals	Increased frontal cortical oscillations
Conen et al. (2015)	-	Remitted MDD and healthy controls	Effects on resting-state activity
Katona et al. (2012)	5 mg/day	Elderly (≥ 65 years) with MDD	Improved cognition (RAVLT, DSST)
McIntyre et al. (2014)	10–20 mg/day	Adults with MDD	Improved cognition (DSST, RAVLT)
Mahableshwarkar et al. (2015)	10–20 mg/day	Adults with MDD	Improved cognition (DSST)
Harrison et al. (2016)	10–20 mg/day	Adults with MDD	Improved cognition across domains
McIntyre et al. (2017)	10–20 mg/day	Working adults with MDD	Improved cognition and work functioning
Chokka et al. (2019)	10–20 mg/day	Working adults with MDD	Improved long-term functioning predicted by cognitive symptoms
McIntyre et al. (2016)	5–20 mg/day	Adults with MDD	Improved cognition (meta-analysis)
Huang et al. (2022)	10–20 mg/day	Adults with MDD	Improved cognition (meta-analysis)
Rosenblat et al. (2015)	5–20 mg/day	Adults with MDD	Improved cognition vs. other antidepressants (meta-analysis)
Baune et al. (2018)	5–20 mg/day	Adults with MDD	Improved cognition (DSST) vs. other antidepressants (network meta-analysis)
Cao et al. (2019)	10–20 mg/day	Adults with MDD	Improved anhedonia (SHAPS)
McIntyre et al. (2021)	5–20 mg/day	Adults with MDD	Improved anhedonia (pooled analysis)
Mattingly et al. (2023)	5–20 mg/day	Adults with MDD	Long-term improvement in anhedonia
Fagiolini et al. (2021)	10–20 mg/day	Adults with MDD and SSRI/SNRI inadequate response	Improved emotional blunting
Baldwin et al. (2016)	5–20 mg/day	Adults with MDD and high anxiety	Improved anxiety symptoms (meta-analysis)
Adair et al. (2023)	5–20 mg/day	Adults with MDD and high anxiety	Improved anxiety symptoms (updated analysis)
Christensen et al. (2022)	10–20 mg/day	Adults with MDD and comorbid GAD	Improved depressive and anxiety symptoms
Almeida et al. (2023)	10–20 mg/day	Adults with MDD and comorbid GAD	Improved depressive and anxiety symptoms in routine practice
Chokka et al. (2019)	10–20 mg/day	Working adults with MDD	Improved work productivity and cognitive symptoms
Jeong et al. (2022)	5–20 mg/day	Adults with depression and Alzheimer’s disease	No significant improvement vs. placebo
Cumbo et al. (2019)	15 mg/day	Elderly with depression and Alzheimer’s disease	Improved cognition and mood vs. other antidepressants
Di Nicola et al. (2022)	10–20 mg/day	Adults with MDD and alcohol use disorder	Improved depressive symptoms
Basurte-Villamor et al. (2022)	Unspecified maintenance dose	Adults with MDD and substance use disorder	Feasible as maintenance treatment
Santos García et al. (2022)	5–20 mg/day	Adults with Parkinson’s disease and MDD	Improved depressive symptoms and cognition
Baldwin et al. (2022)	5–20 mg/day	Adults with MDD and common physical comorbidities	Improved depressive symptoms
Nomikos et al. (2017)	5–20 mg/day	Adults ≥ 55 years with MDD	Effective and well-tolerated
Alvarez et al. (2014)	2.5–20 mg/day	Adults with MDD	Review of efficacy, safety, and tolerability
Al-Sukhni et al. (2015)	5–20 mg/day	Adults with MDD	Review of efficacy, safety, and tolerability, focus on cognition
Kelliny et al. (2015)	5–20 mg/day	Adults with MDD	Overview of primary and secondary literature
Baldwin et al. (2013)	2.5–20 mg/day	Adults with MDD	Pooled analysis of safety and tolerability
Baldwin et al. (2016)	5–20 mg/day	Adults with MDD	Analysis of safety and tolerability from RCTs and open-label extensions
Jacobsen et al. (2015)	10–20 mg/day	Adults with MDD and SSRI-induced sexual dysfunction	Improved sexual functioning vs. escitalopram
Wang et al. (2013)	10–40 mg/day	Healthy adult males	No effect on cardiac repolarization (thorough QT study)
Chen et al. (2015)	10 mg/day	Healthy adults	No effect on pharmacokinetics/dynamics of aspirin and warfarin
Spina et al. (2012)	-	-	Review of clinically significant drug interactions with newer antidepressants
Fagiolini et al. (2024)	10–20 mg/day (oral drops)	Adults with MDD	Clinical benefits, efficacy, and tolerability of slowly titrated oral drops

Abbreviations:

MDD: Major Depressive Disorder

MADRS: Montgomery-Åsberg Depression Rating Scale

RAVLT: Rey Auditory Verbal Learning Test

DSST: Digit Symbol Substitution Test

SHAPS: Snaith-Hamilton Pleasure Scale

GAD: Generalized Anxiety Disorder

SSRI: Selective Serotonin Reuptake Inhibitor

SNRI: Serotonin-Norepinephrine Reuptake Inhibitor

RCT: Randomized Controlled Trial

### Safety and tolerability profile of vortioxetine

Vortioxetine, at doses of 5–20 mg/day, has been shown to be generally safe and well-tolerated in treating MDD, as evidenced by several studies [56–58]. A pooled analysis of ten randomized, double-blind, placebo-controlled, short-term (6–8 weeks) clinical trials revealed that most treatment-emergent adverse events (TEAEs) in MDD patients receiving vortioxetine were mild or moderate in severity (89.3% for vortioxetine vs. 90.5% for placebo) [19]. Nausea, the most common TEAE, was dose-dependent and transient, with a median duration of 10 to 16 days [59]. The most frequent TEAEs (incidence ⩾5%) occurring at twice the frequency of placebo were nausea and vomiting, plateauing at 15 mg/day [13, 60]. Other common antidepressant TEAEs such as headache, dry mouth, dizziness, and insomnia occurred at placebo levels without a dose effect. Suicidal ideation rates were similar between vortioxetine and placebo groups, including patients aged 18–24 years [13, 60]. A meta-analysis in elderly patients (aged 55–88) found vortioxetine effective and well-tolerated, with common side effects including nausea, dizziness, and headache, but no significant impact on biochemical parameters, vital signs, or body weight [55, 60]. Withdrawal rates due to TEAEs ranged from 4.5 to 7.8% for vortioxetine, which were lower than those for venlafaxine (14.2%) and duloxetine (8.8%) [60].

Patients treated with antidepressants often face key tolerability concerns, including sexual function, weight changes, and cardiac/cardiovascular safety:

1. Sexual Dysfunction: TEAEs related to sexual dysfunction were low in patients receiving vortioxetine (1.6–1.8%) compared to placebo (1.0) Moreover, vortioxetine has been shown to improve sexual function in patients with SSRI-induced sexual dysfunction [60, 61].
2. Weight Changes: In short-term treatment, mean weight changes were similar for vortioxetine and placebo, with no significant weight gain or loss observed [60].
3. Cardiac and Cardiovascular Safety: Vortioxetine demonstrated no clinically significant effects on ECG parameters, including QT intervals, in both MDD patients and healthy subjects [59, 60, 62].

The low incidence of these key tolerability concerns with vortioxetine treatment highlights its potential as a well-tolerated antidepressant option for a wide range of patients, including those with specific tolerability concerns or medical comorbidities.

### Drug-drug interactions

Vortioxetine’s favorable cytochrome P450 (CYP) enzyme inhibitory profile reduces the risk of clinically relevant interactions, especially compared to other antidepressants like fluoxetine, paroxetine, duloxetine, and bupropion [21]. Unlike some SSRIs and SNRIs, vortioxetine does not significantly increase bleeding risks when co-administered with warfarin, aspirin, or other anticoagulants. This has been demonstrated in studies evaluating vortioxetine’s effects on the pharmacokinetics and pharmacodynamics of aspirin and warfarin. However, caution is always advisable during co-administration of any medications with potential interactions [63, 64].

Vortioxetine’s minimal impact on CYP enzymes and its low potential for clinically significant drug-drug interactions are important advantages, particularly for patients with comorbid conditions requiring multiple medications. This favorable interaction profile may contribute to improved treatment adherence and reduced risk of adverse events related to polypharmacy.

### Dosage and formulations

A clear dose-response relationship has been observed for vortioxetine in both clinical practice and clinical trials. Vortioxetine 20 mg/day demonstrated significant differences from placebo in improving total depressive symptomatology as early as week two, whereas vortioxetine 10 mg showed separation from placebo only from week four onward. At week eight, the mean change from baseline in MADRS total score was significantly greater for vortioxetine 20 mg/day compared to 10 mg/day. Notably, the incidence of adverse events did not increase in patients receiving vortioxetine 20 mg/day versus 10 mg/day. In flexible-dose studies, 48.0% of patients had their dose increased to 20 mg/day after one week and 64.3% maintained a final dose of 20 mg/day [25].

The administration of vortioxetine at a daily dose of 20 mg provides a faster and more sustained symptomatic improvement compared to the 10 mg/day dose in individuals diagnosed with MDD, without adversely affecting tolerability [25].

In our clinical experience, the most common side effect of vortioxetine is nausea. A slow titration (i.e., reaching the 10 mg dose in 5–10 days) further improves the tolerability profile and reduces the risk of nausea [65]. This can be easily achieved in countries where a liquid formulation is available.

## Conclusions

Vortioxetine can play a key role in the treatment of certain subgroups of depressed patients, such as those with anhedonia, emotional blunting, cognitive dysfunction, and comorbid anxiety. The drug effectively addresses a range of depressive symptoms, including cognitive problems, anhedonia, and emotional blunting. Vortioxetine’s safety and tolerability profile, including relatively low rates of sexual dysfunction and minimal weight effects, enhance its viability for long-term treatment of MDD. Additionally, its limited drug-drug interactions are beneficial for patients treated with other medications. In conclusion, vortioxetine can provide relief across a broad spectrum of symptoms, including anhedonia, emotional blunting, cognitive dysfunction, and comorbid anxiety symptoms, while maintaining a relatively favorable safety profile.

This makes vortioxetine a valuable treatment option for patients with MDD who present with these specific symptom profiles, allowing for a more personalized approach to treatment.

## Data Availability

No datasets were generated or analysed during the current study.

## Abbreviations

MDD: Major Depressive Disorder
MADRS: Montgomery–Åsberg Depression Rating Scale
RAVLT: Rey Auditory Verbal Learning Test
DSST: Digit Symbol Substitution Test
AD: Alzheimer’s disease
SHAPS: Snaith-Hamilton Pleasure Scale
GAD: Generalized Anxiety Disorder
COPD: Chronic Obstructive Pulmonary Disorders
TEAEs: Treatment-Emergent Adverse Events
